# Plasma biomarkers inclusive of α-synuclein/amyloid-beta40 ratio strongly correlate with Mini-Mental State Examination score in Parkinson’s disease and predict cognitive impairment

**DOI:** 10.1007/s00415-022-11287-5

**Published:** 2022-07-25

**Authors:** Daniel Kam Yin Chan, Jack Chen, Ren Fen Chen, Jayesh Parikh, Ying Hua Xu, Peter A. Silburn, George D. Mellick

**Affiliations:** 1 University of New South Wales, Sydney, Australia; 2 NICM Health Research Institute, Western Sydney University, Sydney, Australia; 3 Bankstown-Lidcombe Hospital, Eldridge Rd,, Bankstown, NSW 2200 Australia; 4 Central Sydney Immunology Laboratory at Royal Prince Alfred Hospital, Sydney, NSW Australia; 5 Queensland Brain Institute, University of Queensland, Brisbane, QLD Australia; 6 Griffith University, Brisbane, QLD Australia

**Keywords:** Parkinson’s disease, Biomarkers, α-synuclein, Amyloid-beta40, Mini-Mental State Examination score, Cognitive impairment

## Abstract

Plasma biomarkers for Parkinson’s disease (PD) diagnosis that carry predictive value for cognitive impairment are valuable. We explored the relationship of Mini-Mental State Examination (MMSE) score with plasma biomarkers in PD patients and compared results to vascular dementia (VaD) and normal controls. The predictive accuracy of an individual biomarker on cognitive impairment was evaluated using area under the receiver operating characteristic curve (AUROC), and multivariate logistic regression was applied to evaluate predictive accuracy of biomarkers on cognitive impairment; 178 subjects (41 PD, 31 VaD and 106 normal controls) were included. In multiple linear regression analysis of PD patients, α-synuclein, anti-α-synuclein, α-synuclein/Aβ40 and anti-α-synuclein/Aβ40 were highly predictive of MMSE score in both full model and parsimonious model (*R*^2^ = 0.838 and 0.835, respectively) compared to non-significant results in VaD group (*R*^2^ = 0.149) and in normal controls (*R*^2^ = 0.056). Α-synuclein and anti-α-synuclein/Aβ40 were positively associated with MMSE score, and anti-α-synuclein, α-synuclein/Aβ40 were negatively associated with the MMSE score among PD patients (all *P*s < 0.005). In the AUROC analysis, anti-α-synuclein (AUROC = 0.788) and anti-α-synuclein/Aβ40 (AUROC = 0.749) were significant individual predictors of cognitive impairment. In multivariate logistic regression, full model of combined biomarkers showed high accuracy in predicting cognitive impairment (AUROC = 0.890; 95%CI 0.796–0.984) for PD versus controls, as was parsimonious model (AUROC = 0.866; 95%CI 0.764–0.968). In conclusion, simple combination of biomarkers inclusive of α-synuclein/Aβ40 strongly correlates with MMSE score in PD patients versus controls and is highly predictive of cognitive impairment.

**Supplementary Information:**

The online version contains supplementary material available at 10.1007/s00415-022-11287-5.

## Introduction

Parkinson’s disease (PD) is a progressive neurodegenerative disease that ultimately results in disabling physical and cognitive impairments [1]. Recognizing the importance of the emergence of cognitive impairment and dementia in PD, the Movement Disorder Society has recommended Mini-Mental State Examination (MMSE) as a Level 1 clinical tool to assess PD associated with a decreased global cognitive efficiency [2]. Further validation was subsequently carried out by Ohta et al. [3] in a large population study and found MMSE to be a useful screening tool.

While MMSE scores may be a useful clinical tool for detecting cognitive impairment in PD, it remains to be seen if the MMSE scores bear any relationship with diagnostic biomarkers of PD that can ultimately predict the occurrence of cognitive impairment or dementia. Such a proof-of-concept study has clinical significance since an adverse level of biomarkers may alert clinicians of the higher risk of cognitive decline in PD patients ahead of time.

Interestingly, a recent 99 m Tc-TRODAT-1 imaging study found that, when compared with normal adults, PD patients with cognitive impairment have higher plasma levels of the biomarkers α-synuclein and T-tau, and lower levels of amyloid-beta40 (*P* < 0.05) [4]. Our recent study [5] found the ratios of α-synuclein/amyloid-beta40 (α-synuclein/Aβ40) and anti-α-synuclein/amyloid-beta40 (anti-α-synuclein/Aβ40) have high specificities in predicting PD in two independent patient samples (total *n* = 272) with 98% in the training set and 90.5% in in the validation set. However, whether the aforementioned ratios also predict cognitive impairment (MMSE scores) in PD is unclear. This approach is novel as plasma α-synuclein may be regarded as a predictive risk factor for the development of cognitive impairment in PD while Aβ40 may be protective, and the respective ratios between them could be a valuable predictor for the development of cognitive impairment/dementia in PD.

Furthermore, dementia of PD (PDD) is commonly associated with Alzheimer’s disease (AD), with 28.6% of all PD cases shown to have sufficient pathology for comorbid AD [6]. The abnormal aggregation of Aβ42 in plaques is a salient feature of AD [7], and the reduction in plasma Aβ42 may be a marker for AD status [8].

Taking these preliminary data together, we hypothesize that a relative increase in plasma α-synuclein or anti-α-synuclein to amyloid-beta40 (Aβ40) in conjunction with a decreased level of Aβ42 may reflect the development of cognitive impairment (or dementia) in PD. If the magnitude of the aforementioned ratios of biomarkers bears relationship with the magnitude in MMSE scores, the markers could be valuable surrogates for the staging of PD. Although such a relationship is best demonstrated in a longitudinal study, recruiting a cohort and measuring MMSE scores over time to evaluate significant decline may take years. Hence, a cross-sectional study as a proof-of concept pilot is a more practical approach to investigate if biomarker ratios mentioned before correlate with the MMSE scores and if they can also predict cognitive impairment.

### Aim

To investigate the relationship of MMSE score (and cognitive impairment) with the plasma biomarkers: α-synuclein, anti-α-synuclein and their respective ratios to amyloid-beta40 (Aβ40), along with amyloid-beta42 (Aβ42) in Parkinson’s disease subjects.

## Methods

### Subjects’ recruitment, settings, diagnoses of PD and MMSE assessment

Parkinson’s disease subjects and controls were recruited from 2 centres, as part of the Queensland Parkinson’s Project [9] and the Bankstown-Lidcombe Hospital vascular dementia and healthy longevity project [10]. Additional Parkinson’s disease subjects were recruited from Bankstown-Lidcombe Hospital as part of the biomarkers and Parkinson’s disease project [5]. Further details of sampling and diagnostic methodology could be obtained from previous studies [5, 9, 10].

Briefly, all subjects were assessed by movement disorder specialists or geriatricians/neurologists with expertise in movement disorders. The Parkinson’s disease diagnostic criteria of Gelb et al. [11] were used for the Queensland project, and a more updated criteria endorsed by Movement Disorders Society was used for Bankstown-Lidcombe study [12]. The accuracy of the diagnosis had been reinforced with continuous review during patients’ follow-up, and any correction of diagnoses were updated.

Thirty-one subjects with the diagnosis of small vessel vascular dementia (VaD) were used as the first control group (cases with dementia but not PD). The ascertainment of the diagnosis of dementia was explained in the previous study [10]. The second control group (without dementia or PD) consisted of one hundred and six older subjects with MMSE scores of ≥ 28/30 [10]. All were examined and cases excluded if there was any concern of dementia or Parkinsonism. All participants in both groups were assessed with MMSE scores.

### MMSE assessment

All researchers performing MMSE were trained, and examinations were done at the time that plasma samples were collected. Cases that were delirious, with untreated depression, could not communicate effectively with examiners due to language or dysphasia or who did not attend any schooling were excluded from the current study. The MMSE was conducted as a routine for the “healthy longevity and vascular dementia” project and for PD subjects who have complaints of cognitive symptoms. Cognitive impairment was pragmatically defined as having an MMSE score < 27.

#### Inclusion and exclusion criteria

Diagnosis of PD cases was based on the presence of bradykinesia and another feature of tremor or rigidity. All PD cases with MMSE scores were eligible for further consideration in analysis but were excluded if cases have atypical features for PD [12] or if assessment of MMSE was affected by aforementioned reasons such as no education or language barrier.

All controls were screened for PD and those with possible PD diagnosis or Parkinsonism were excluded. A control group with small vessel vascular dementia was also assessed for dementia diagnosis [10] while the control group without dementia (normal controls) was examined for any evidence of cognitive impairment [10].

### Laboratory methods

ELISAs were used as per protocols previously explained for plasma biomarkers α-synuclein, anti-α-synuclein, Aβ40 and Aβ42 [5, 13–15]. Given the fact that plasma α-synuclein measurement can vary if collection and laboratory method is not optimal, we have taken specific caution to mitigate spurious results which were described in previous publication [5]. Further details are also included in supplementary materials.

### Ethics

Ethics approval was obtained from SWSLHD (2019/ETH04525) and Griffith University (ESK/04/11/HREC).

### Statistical analysis

We provided median and interquartile range for continuous distributed variables after inspection of their distributions across the three groups (PD, VaD control and normal control). The group differences of continuous variables were tested using Wilcoxon rank sum test with exact probability. To understand the relationship between MMSE score and a combination of biomarkers and other demographic variables, we used multiple linear regression with both forced entry method and backward elimination with probability of removing from the model set at 0.10. The biomarkers in the model included α-synuclein, anti-α-synuclein, Aβ40, Aβ42, the ratios of α-synuclein/Aβ40 and anti-α-synuclein/Aβ40. To avoid the numerical problem associated with different scales of these biomarkers, the value of α-synuclein/Aβ40 and anti-α-synuclein/Aβ40 was multiplied by 10^3^. The coefficient of determination (*R*^2^) was used to assess the overall predictive power of combined biomarkers. We compared the individual predictors and overall *R*^2^ on MMSE score across the three groups. To understand predictive accuracy of different biomarkers on cognitive impairment, we firstly produced area under the receiver operating characteristic curve (AUROC) for each individual biomarker among two comparison groups: PD vs control and VaD vs control, respectively. A multivariate logistic regression model was then used to evaluate the predictive accuracy of combined biomarkers on cognitive impairment of the two comparison groups, respectively. Similar to multiple linear model, both forced entry and backward elimination method were used and model-based AUROC generated. We also reported sensitivity, specificity, total correct classification, positive likelihood ratio (LR +) and negative likelihood ratio (LR−) for selected cut-off points based on the combination of biomarkers. A *P* value of 0.01 was considered as statistically significant, and all analyses were conducted in Stata™ (Stata Statistical Software: Release 17. College Station, TX: StataCorp LLC; 2021).

## Results

### The distribution of age, biomarkers and MMSE score across three groups

A total of 43 PD cases with concomitant MMSE scores were eligible for consideration in the study. After excluding 2 PD cases due to MMSE ineligibility (one with communication issue due to language barrier and another was uneducated), 41 PD patients remained for analysis. The median age was 67 years old (IQR: 13 years). All 31 cases with vascular dementia and 106 normal controls all had MMSE and were included for analysis. Details of the distribution of variables are summarized in Table 1. There were statistically significant differences between the PD and control groups on all biomarkers (Table 1). There were also significant differences in α-synuclein and anti-α-synuclein, respectively, between the VaD group and normal control group.

**Table 1:** Descriptive results for the continuous distributed variables (median, IQR) by groups

	PD (*n* = 41)			VaD (*n* = 31)			Control (*n* = 106)		Total (*n* = 178)	
	Median	IQR	*P*	Median	IQR	*P*	Median	IQR	Median	IQR
Age	67.000	13.000	< 0.001	83.000	6.000	0.328	83.000	5.000	82.000	8.000
α-synuclein	1.515	1.701	< 0.001	0.659	1.031	0.008	0.444	0.565	0.578	0.907
anti-α-synuclein	4.734	4.938	< 0.001	2.255	3.361	0.001	1.260	1.897	1.703	2.674
Aβ40	52.367	56.066	0.011	51.678	70.207	0.253	42.643	65.393	48.643	65.879
Aβ42	32.961	21.268	0.019	73.229	117.012	0.028	45.634	70.316	42.532	65.150
α-synuclein/Aβ40	0.022	0.037	0.010	0.013	0.004	0.384	0.011	0.016	0.012	0.015
anti-α-synuclein/Aβ40	0.093	0.109	< 0.001	0.041	0.015	0.054	0.027	0.035	0.032	0.039
MMSE	28.000	3.000	< 0.001	20.000	6.000	< 0.001	30.000	1.000	29.000	2.000

*P* values from Wilcoxon rank sum test with exact probability for PD vs control and VaD vs control group, respectively. To avoid the numerical problem associated with different scales of these biomarkers, α-synuclein/Aβ40 and anti-α-synuclein/Aβ40 were multiplied by 10^3^

### The multiple linear regression results of biomarkers’ predicting accuracy on MMSE score

The significance of individual biomarkers and *R*^2^ of regression models for all three groups are presented in Table 2. In the PD group, biomarkers α-synuclein, anti-α-synuclein, α-synuclein/Aβ40 and anti-α-synuclein/Aβ40 were highly significant in a full model and parsimonious model: with *R*^2^ = 0.838 and 0.835, respectively, compared to non-significant results in the VaD group (*R*^2^ = 0.149) and in the normal control group (*R*^2^ = 0.056). None of the individual biomarkers was a significant predictor for MMSE score in both the VaD control group and the normal control group. Α-synuclein and anti-α-synuclein/Aβ40 were positively associated with MMSE score, and anti-α-synuclein and α-synuclein/Aβ40 were negatively associated with the MMSE score among PD patients (all *P*s < 0.005; Table 2).

**Table 2:** Predictive value of biomarkers and age on MMSE score

	MMSE (PD group-full model)			MMSE (PD group-parsimonious model)			MMSE (VaD)			MMSE (control)		
	*b*	95% CI	*P* value	*b*	95% CI	*P* value	*b*	95% CI	*P* value	*b*	95% CI	*P* value
α-synuclein	2.007***	(1.358–2.656)	0.000	2.005***	(1.386–2.624)	0.000	− 43.145	(− 273.348–187.057)	0.702	0.290	(− 0.326–0.906)	0.352
anti-α-synuclein	− 0.726***	(− 1.086–0.366)	0.000	− 0.681***	(− 0.942 to − 0.420)	0.000	6.122	(− 37.950–50.195)	0.777	− 0.107	(− 0.291–0.076)	0.249
Aβ40	0.005	(− 0.017–0.027)	0.642							− 0.015	(− 0.032–0.003)	0.100
Aβ42	0.008	(− 0.026–0.042)	0.640				0.192	(− 0.547–0.931)	0.597	0.012	(− 0.003–0.028)	0.118
α-synuclein/Aβ40	− 0.089***	(− 0.104–0.073)	0.000	− 0.090***	(− 0.104–0.076)	0.000	1.542	(− 13.224–16.308)	0.831	− 0.005	(− 0.013–0.002)	0.172
anti-α-synuclein/Aβ40	0.027**	(0.009–0.046)	0.005	0.025***	(0.013–0.037)	0.000	− 0.184	(− 2.885–2.517)	0.890	0.001	(− 0.000–0.003)	0.120
Age	0.025	(− 0.048–0.098)	0.486				0.061	(− 0.253–0.374)	0.693	− 0.008	(− 0.036–0.020)	0.584
Intercept	26.726***	(20.547–32.904)	0.000	29.126***	(28.046–30.206)	0.000	3.868	(− 71.185–78.921)	0.916	30.112***	(27.741–32.484)	0.000
*N*		41			41			31			106	
*R*^2^		0.838			0.835			0.149			0.056	

A parsimonious model was based on backward elimination with probability removed from the model set as 0.10; for control group and VaD group, a parsimonious model only produced intercept only. We also initially included duration of PD diagnosis and gender in the full model but did not find them as significant predictors, and therefore, we excluded them from further analyses for simplicity. To avoid the numerical problem associated with different scales of these biomarkers, α-synuclein/Aβ40 and anti-α-synuclein/Aβ40 were multiplied by 10^3^

*P < 0.05; **P < 0.01; ***P < 0.001

### *The AUROC analyses of both individual and combined biomarkers on prediction of cognitive impairment (MMSE* < *27) in PD and VaD groups*

The AUROC analyses of individual biomarkers for predicting cognitive impairment in PD and VaD versus normal controls are presented in Table 3. Anti-α-synuclein (AUROC = 0.788) and anti-α-synuclein/Aβ40 (AUROC = 0.749) were the only significant predictors of cognitive impairment. Age was not a significant predictor.

**Table 3:** AUROC analyses of individual biomarkers and age for predicting cognitive impairment in Parkinson’s disease (PD) and small vessel dementia (VaD)

	PD + Control (*n* = 147)		VaD + Control (*n* = 137)	
	AUROC	95%CI	AUROC	95%CI
α-synuclein	0.662	(0.312–1.000)	0.655	(0.537–0.773)
anti-α-synuclein	0.788	(0.593–0.984)	0.687	(0.572–0.802)
Aβ40	0.515	(0.263–0.767)	0.568	(0.460–0.676)
Aβ42	0.339	(0.145–0.533)	0.629	(0.513–0.746)
α-synuclein/Aβ40	0.605	(0.249–0.961)	0.552	(0.459–0.644)
anti-α-synuclein/Aβ40	0.749	(0.575–0.924)	0.614	(0.515–0.713)
Age	0.171	(0.085–0.258)	0.442	(0.324–0.560)

The predictive accuracy of combined biomarkers from the logistic regression model is presented in Table 4 (also included details of models M1–M4). For PD plus controls, a full model based on forced entry method (M1) showed highly predictive accuracy (AUROC 0.890; 95%CI 0.796–0.984) which was comparable to a parsimonious model (M2; AUROC 0.866; 95%CI 0.764–0.968). The AUROCs based on combination of different biomarkers were significantly better than any individual biomarker. Anti-α-synuclein (*b* = 0.186; *P* = 0.048) was borderline significant, and α-synuclein/Aβ40 (*b* = 0.015; *P* = 0.006) was the only significant biomarker for predicting cognitive impairment in PD group (M2; Table 4). For VaD plus normal controls, α-synuclein and Aβ42 were positive predictors and Aβ40, α-synuclein/Aβ40 were negative predictors in both the full model (M3: AUROC = 0.935) and the parsimonious model (M4: AUROC = 0.931; Table 4).

**Table 4:** AUROC analyses of combined biomarkers and age for predicting cognitive impairment in PD group and VaD group (the four models M1–M4)

	M1		M2		M3		M4	
	*b*	*P*	*b*	*P*	*b*	*P*	*b*	*P*
α-synuclein	− 0.079	0.898			12.157*	0.033	13.479**	0.007
anti-α-synuclein	0.224	0.189	0.186*	0.048	0.251	0.855		
Aβ40	0.019	0.176			− 0.545**	0.003	− 0.561**	0.002
Aβ42	− 0.023	0.261			0.247**	0.009	0.253**	0.005
α-synuclein/Aβ40	0.022	0.076	0.015**	0.006	− 0.426*	0.010	− 0.473**	0.003
anti-α-synuclein/Aβ40	− 0.004	0.453			− 0.016	0.614		
Age	− 0.002	0.976			− 0.060	0.342		
Intercept	− 4.591	0.436	− 4.52***	0.000	12.583*	0.023	7.694**	0.001
AUROC	0.890		0.866		0.935		0.931	
*N*	147		147		137		137	

M1 = full model for PD group; M2 = parsimonious model for PD group; M3 = full model for VaD; M4 = parsimonious model for VaD. To avoid the numerical problem associated with different scales of these biomarkers, α-synuclein/Aβ40 and anti-α-synuclein/Aβ40 were multiplied by 10^3^

*P < 0.05; **P < 0.01; ***P < 0.001

**Fig. 1:**
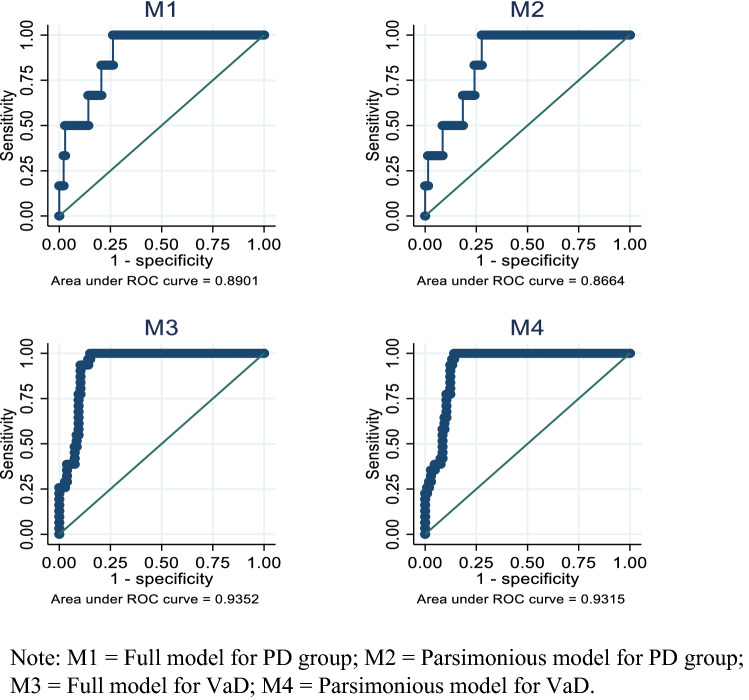
ROC of Model 1 to Model 4 (M1–M4)

The overall receiver operating characteristics curve (ROC) for M1–M4 is presented in Fig. 1. The sensitivity and specificity of M1–M4 in predicting cognitive impairment at a chosen cut-off value of combined biomarkers are presented in Table 5. Based on an index generated from logistic regression modelling results, if the index of M2 ≥ − 3.65 is used, a sensitivity of 100% and specificity of 72.3% are achieved, with 73.5% of cases correctly classified. If the index of M4 ≥ − 0.852 is used, the sensitivity is 100% and specificity 85.9% and 89.1% are correctly classified (Table 5).

**Table 5:** Sensitivity and specificity of M1–M4 in predicting cognitive impairment at a chosen cut-off values of combined biomarkers

Cut point	Sensitivity (%)	Specificity (%)	Correctly classified (%)	LR +	LR−
M1: ≥ − 3.81	100.00	73.76	74.83	3.811	0.000
M2: ≥ − 3.65	100.00	72.34	73.47	3.615	0.000
M3: ≥ − 1.15	100.00	84.91	88.32	6.625	0.000
M4: ≥ − 0.852	100.00	85.85	89.05	7.067	0.000

M1 = full model for PD group; M2 = parsimonious model for PD group; M3 = full model for VaD; M4 = parsimonious model for VaD. For M1–M4: an index was generated, respectively, based on the logit model results as shown in Table 4. LR + : positive likelihood ratio; LR−: negative likelihood ratio

## Discussion

Our study found that there were significant differences in the distributions of a group of biomarkers between PD patients and controls, and between small vessel vascular dementia patients and controls. We found significantly high predictive accuracy of a group of biomarkers (α-synuclein, anti-α-synuclein, α-synuclein/Aβ40 and anti-α-synuclein/Aβ40) on MMSE score among PD patients but not in vascular dementia patients and normal controls. Furthermore, different combinations of biomarkers can accurately predict cognitive impairment (MMSE < 27) both in PD patients and in patients with small vessel vascular dementia. We also found an index based on combination of biomarkers outperformed any individual biomarker and could predict cognitive impairment with great accuracy.

A biomarker that can accurately diagnose disease early is good and more valuable if it can provide additional information on disease severity or staging. However, finding a single biomarker that possesses these properties has been elusive probably because of the complex multifactorial nature of PD. MMSE score decline is a reflection of cognitive impairment and dementia of PD and is therefore a useful clinical indicator of disease progression as most PD subjects who do not have dementia in initial presentation would have normal MMSE score. However, MMSE score can also decline with the onset of Alzheimer’s disease; therefore, a panel of plasma biomarkers (including Aβ42) that can predict progression if such scenario occurs will have clinical utility. We are not aware of any composite plasma biomarkers published thus far that have yielded consistent results on the dual qualities of early diagnosis and disease progression.

**Fig. 2:**
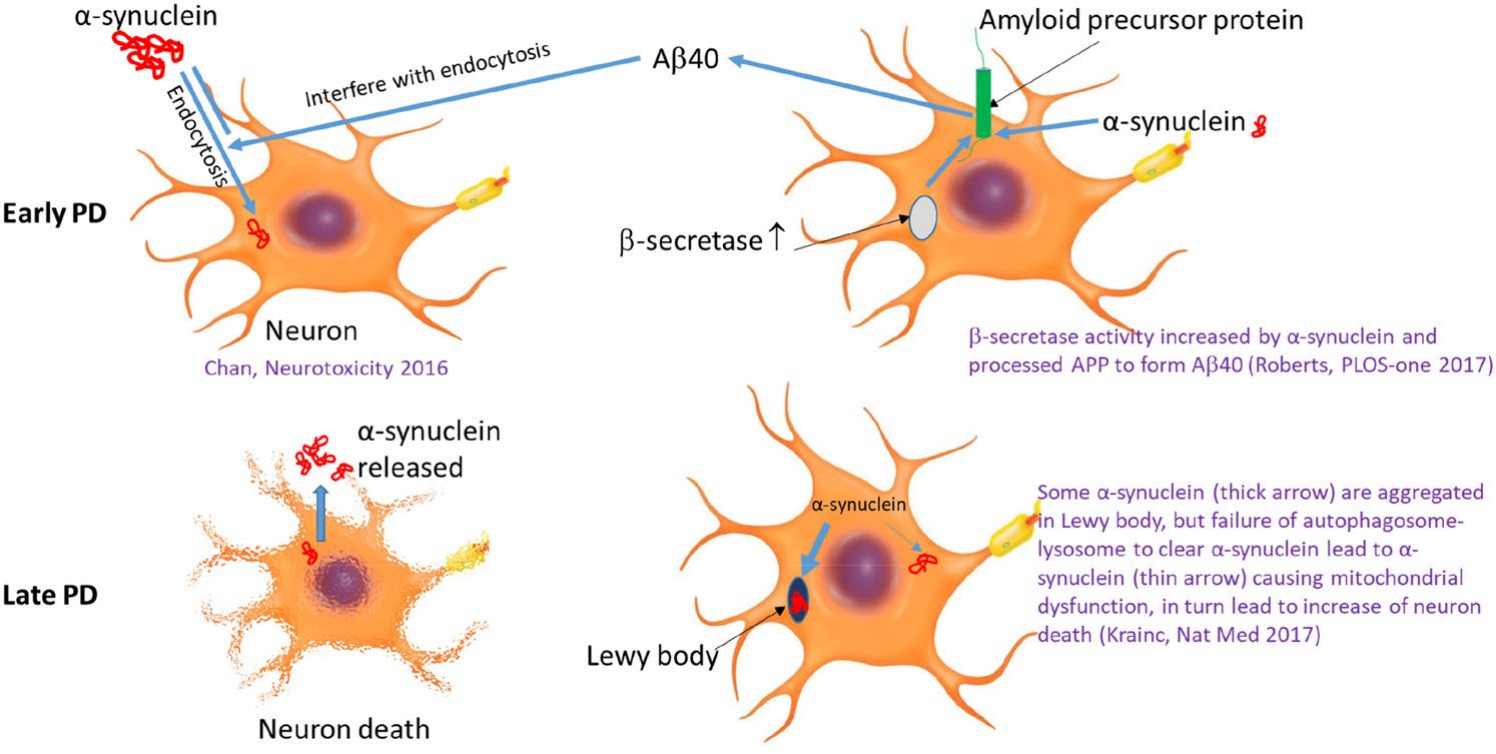
Schematic diagram explaining the relationships of α-synuclein and Aβ40 at cellular level

Our finding of the significant inverse relationship between the α-synuclein/Aβ40 ratio and MMSE score in PD subjects is worthy of note. It also contrasts with the lack of correlation in the two other groups (normal controls and VaD). Therefore, the ratio α-synuclein/Aβ40 may be valuable for predicting cognition decline (MMSE score) in PD subjects and a low ratio may be a protective predictor against poor cognition (low MMSE score). This proposition is consistent with our recent report that a high ratio of α-synuclein/Aβ40 is highly specific for PD diagnosis including early PD (duration < 5 years) [5]. Our finding also echoed that of Chen et al. [4]. Their study found that the MMSE score was significantly related to increased level of α-synuclein and decreased level of Aβ40 in PD subjects with impaired cognition. Adding weight to these observations is the post-mortem study by Love et al. which found statistical significant increase in soluble α-synuclein with concomitant decrease in soluble Aβ40 in subjects with PD dementia compared to controls in 3 out of 4 brain regions [16]. Recent studies have found α-synuclein toxicity and many cellular functions such as autophagosome–lysosome system and mitochondria are intimately related [17]. The α-synuclein toxicity in PD can result in neuronal deaths and cognitive impairment (Fig. 2).

Taking it further, the notion of the importance of α-synuclein/Aβ40 ratio in predicting disease progression is supported by a previous study that found Aβ40 (not Aβ42) in vitro reduced the neuronal uptake of α-synuclein by 80% [18]. The decrease in neuronal uptake of α-synuclein was probably related to the interference of endocytosis of α-synuclein by Aβ40 [18]. A drop in the levels of soluble Aβ40 due to cell death as PD progresses in relevant brain regions may manifest itself with the clinical complication of cognitive impairment and dementia. This would explain why Aβ40 level drops when cognitive impairment occurs in late PD. The complex function of Aβ40 in relation to α-synuclein is depicted in Fig. 2.

Our PD subjects are broadly representative of community setting with a wide range of MMSE score (6–30) and age distribution (median age 67 years old, inter-quartile range 13). Furthermore, our PD patients had relatively higher plasma levels of Aβ40 compared to the controls, but the rise in levels compared to controls is not as much as plasma α-synuclein. Hence, a relative increase in plasma α-synuclein to Aβ40 may predict risks of PD. This is consistent with the study of Roberts et al. that α-synuclein enhances cellular processing of amyloid precursor protein by β-secretase in vitro [19]. However, the up-regulation of Aβ40 production is probably halted in late PD due to neuronal dysfunction or cell deaths, caused by increased α-synuclein uptake by neurons (see illustration in Fig. 2), hastening the process of cognitive impairment/dementia.

Compared to the result of the 3 individual plasma biomarkers (Aβ40, α-synuclein and T-tau) used to discriminate PD patients with cognitive impairment versus controls in the study of Chen et al. [4] with sensitivity in the nineties and specificity in the seventies, our parsimonious M2 model (α-synuclein/Aβ40 and anti-α-synuclein) performed just as well (Table 5). The equally good performance of our model adds confidence to the validity of our approach.

Interestingly, we found a combination of plasma biomarkers (including α-synuclein, Aβ40, Aβ42 and α-synuclein/Aβ40) produces good prediction of cognitive impairment in VaD (Table 4, M3 and M4), but not individual biomarker. One possible explanation could be that patients with small vessel vascular dementia might have incidental comorbid pathologies including that of Alzheimer’s disease and PD, but discerning such comorbid diagnoses might be clinically difficult. Hence, patients were classified clinically as VaD only when it fact they possibly have the aforementioned pathologies. Therefore, when these plasma biomarkers (for testing PD and AD) were combined for predicting cognition, it yielded significant predictive result for cognitive impairment compared to individual biomarker due to this reason.

Another interesting finding was that although we included duration of PD and gender in our initial model when analysing correlation with MMSE score, they were not found to be significant predictors and were therefore excluded from further analyses (Table 2). This may imply the biomarkers (including the ratios) included in final modelling were more reliable independent predictors for MMSE score (or cognitive decline) than duration of PD or gender. The importance of possible cognitive decline in PD in the long-term is relevant, but there is a dearth of longitudinal studies examining this crucial matter. A recent systematic review found only 5 studies with 4 years’ duration or more that studied cognitive decline in PD and few included comprehensive neuropsychological assessment [20].

Finally, our study is limited by small sample size, but we do hope the results of this proof-of-concept pilot can be replicated in longitudinal studies with bigger sample size or in other settings.

## Conclusion

Our finding that the ratio of plasma α-synuclein/Aβ40 had predictive value which correlated well with MMSE score and cognitive impairment in PD is novel and worthy of further studies. If replicated in larger cohorts, it will also add weight to the proposition that Aβ40 is protective against PD progression and could functionally interfere with the endocytosis of α-synuclein. This could explain why a low ratio is protective against the decline of MMSE score (PD progression) and a high ratio predicts cognitive impairment. Future studies into whether Aβ40 or equivalent peptides may have therapeutic values in modifying the progression of PD will be of interest.

## Supplementary Information

Below is the link to the electronic supplementary material.

Supplementary file1 (DOCX 13 KB)
