# Supplementary material for: Plasma biomarkers inclusive of α-synuclein/amyloid-beta40 ratio strongly correlate with Mini-Mental State Examination score in Parkinson’s disease and predict cognitive impairment
Source: J Neurol. 2022 Jul 25;269(12):6377–85. doi: 10.1007/s00415-022-11287-5 (PMC9618522; doi:10.1007/s00415-022-11287-5)
Supplement: Supplementary file 1 — Supplementary file1 (DOCX 13 KB) [file 415_2022_11287_MOESM1_ESM.docx]

**Supplementary materials**

**Detection of α‑Synuclein Levels**

Plasma α-synuclein levels were measured as previously described by Ishii et al. [13] with some modifications. Briefy, the ELISA plate (Nunc Maxisorb, NUNC, Denmark) was coated with 1 μg/ml of anti-human α-synuclein monoclonal antibody 211 (Santa Cruz Biotechnology, USA) (100 μl/well) in 200 mM NaHCO3 (Sigma–Aldrich, USA), pH 9.6, containing 0.02% (w/v) sodium azide, washed four times with PBST (phosphate buffered saline containing 0.05% Tween 20), and incubated with 200 μl/well of blocking buffer (PBS containing 2.5% gelatin and 0.05% Tween 20) for 2 h. After washing with PBST, 100 μl of the sample diluted with heterophilic antibody inhibitor (ELISA diluent, MABTECH, Sweden) was added to each well, and incubated at 37°C for 3 h. α-synuclein was detected using 0.2 μg/ml of FL-140 antibody (100 μl/well) diluted to 1:1000 in blocking buffer, followed by incubation with 100 μl/well (1:10,000 dilution) of HRP-labelled anti-rabbit antibody (DAKO, Denmark). A relative concentration estimate of total α-synuclein in the samples was calculated according to the standard curve, which was carried out in each ELISA plate using 100 μl/ well of recombinant human α-synuclein (rPeptide, USA) solution at different protein concentrations in PBS.

**Detection of Anti‑α‑Synuclein Auto‑antibodies**

Anti-α-synuclein auto-antibodies were measured as previously described [14] with some modifications. Initially, 100 µl/well of 1 µg/ml α-synuclein in phosphate buffered saline (PBS) buffer was incubated overnight in 96-well plates (Maxisorp, Nunc), followed by 1 h blocking with 200 µl/well of PBS-T containing 1% BSA (Sigma) at RT. After washing with 200 µl PBS, 100 µl of diluted specimen was applied on the plates. The initial dilution for samples was 20-fold in PBS. This was followed by twofold serial dilution steps in PBS, keeping the volumes of all samples at 100 µl. After 3 h incubation, the plates were washed 3 times with 200 µl PBS Tween (0.1%). Afterwards, the plates were covered with horse radish peroxidase labelled antihuman IgG antibodies in PBS-Tween (20000x dilution, A8792, Sigma) and incubated for 1 h. After washing away unbound secondary antibody, the detection was performed by using an ECBlue Enhanced TMB substrate (10-9405, Medicago). The reaction was stopped by adding 1 M sulfuric acid and absorbance was measured at 450 nm on a Fluorostar plate reader.

**Detection of Aβ40**

Human Aβ40 in samples were detected using an established ELISA as previously described in the manufacturer’s instructions modified from well-established methods [15]. Briefly, the assay uses a specific Aβ40 antibody pre-coated in the wells of the supplied microplate. Samples, standards, or controls are then added into these wells and bound to the immobilized (capture) antibody. The sandwich is formed by the addition of the second (detector) antibody; a substrate solution is added that reacts with the enzyme-antibody-target complex to produce measurable signal. The intensity of this signal is directly proportional to the concentration of Aβ40 present in the plasma.
